# Mirizzi syndrome type V complicated with triple fistula: a case report

**DOI:** 10.1186/s40792-023-01696-7

**Published:** 2023-06-19

**Authors:** Miltiadis Lalountas, Nikolaos Smyrlis, Stylianos Vladimiros Mouratidis, Panagiotis Makedos

**Affiliations:** 1 Department of Surgery, General Hospital of Polygyros, Chalkidiki, Greece; 2 54249 Thessaloniki, Greece

**Keywords:** Mirizzi syndrome, Triple fistula, Case report

## Abstract

**Background:**

Mirizzi syndrome (MS) is a complicated form of longstanding, symptomatic cholelithiasis. According to Beltran Classification MS Type V has been introduced to describe the cholecystoenteric fistula, with or without gallstone ileus. Mirizzi syndrome Type V with double fistula has been reported in the past; however, the triple fistula is an even rarer case, first described in the international literature so far.

**Case presentation:**

A 77-year-old male was admitted to our surgical department with recurrent episodes of abdominal pain, which initially presented in the last 6 months and was accompanied with jaundice. Computed tomography showed findings of cholelithiasis, pneumobilia and choledocholithiasis. We performed an ERCP, which showed two fistulas of the gallbladder with the pyloric antrum and the duodenum, respectively. Surgical treatment was immediately undergone and during laparotomy, we confirmed these findings. We ligated and dissected these communications. In addition, a third fistula between the gallbladder and the common bile duct was identified. An insertion of a Kehr T-tube into the common bile duct was performed via the gallbladder. After 3 months, the Kehr T-tube was removed and in the subsequent 2 years of follow-up the patient was presented without complications.

**Conclusions:**

Mirizzi syndrome complicated with triple fistula, first described in the international literature, to the best of our knowledge, confirms the long natural history of inflammation.

## Background

Mirizzi syndrome (MS) is a rare condition caused by the obstruction of the common bile duct (CBD) or common hepatic duct (CHD) by external compression from multiple impacted gallstones or a single large impacted gallstone in Hartman's pouch [1]. This syndrome is a complication of prolonged cholelithiasis, with prevalence from 0.05% to 2.7% among patients with calculi of the gallbladder. It presents a spectrum that varies from extrinsic compression of the CHD to the presence of cholecystobiliary fistula [2]. Usually, this syndrome is associated with cholecystobiliary fistula (cholecystohepatic or cholecystocholedochal), as in type II or III according to Csendes et al. classification, but the presence of an internal biliary fistula as cholecystoduodenal, cholecystocolonic or cholecystogastric requires the most recent Beltran Classification, in which Type V has been introduced to describe the cholecystoenteric fistula, with or without gallstone ileus [3, 4]. MS Type V with double fistula has been reported in the past; however, the triple fistula is an even rarer case first described in the international literature so far.

## Case presentation

A 77-year-old male was admitted to our surgical department with recurrent episodes of abdominal pain, accompanied with jaundice. The symptoms were initially presented in the last 6 months before he visited our hospital. The patient denied any clinical symptoms before the last 6 months. These findings led us to perform an abdominal computed tomography (CT) to diagnose the cause of this clinical presentation. The examination showed findings of cholelithiasis, pneumobilia and choledocholithiasis.

**Fig. 1:**
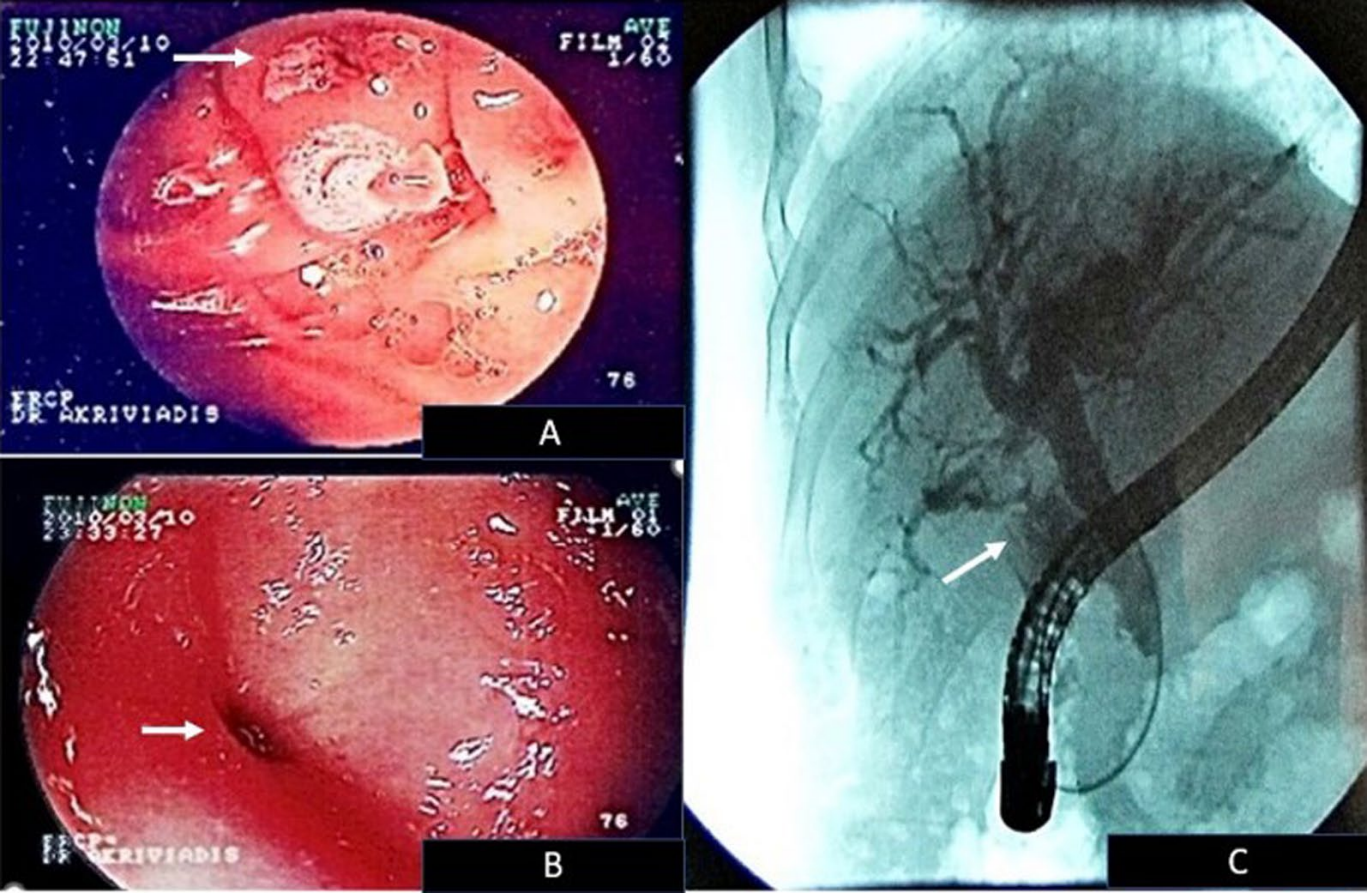
ERCP findings of **A** a cholecystoduodenal fistula that is located above the ampulla of Vater (arrow), **B** a fistula between the gallbladder and the pyloric antrum (arrow) and **C** dilation of the biliary tree with contrast escape into the intestine, as a result of the communication with the gastrointestinal tract (arrow)

The CT findings led us to perform an endoscopic intervention and an endoscopic retrograde cholangiopancreatography (ERCP) was carried out. It showed two fistulas of the gallbladder with the pyloric antrum and the duodenum, respectively (Fig. 1).

**Fig. 2:**
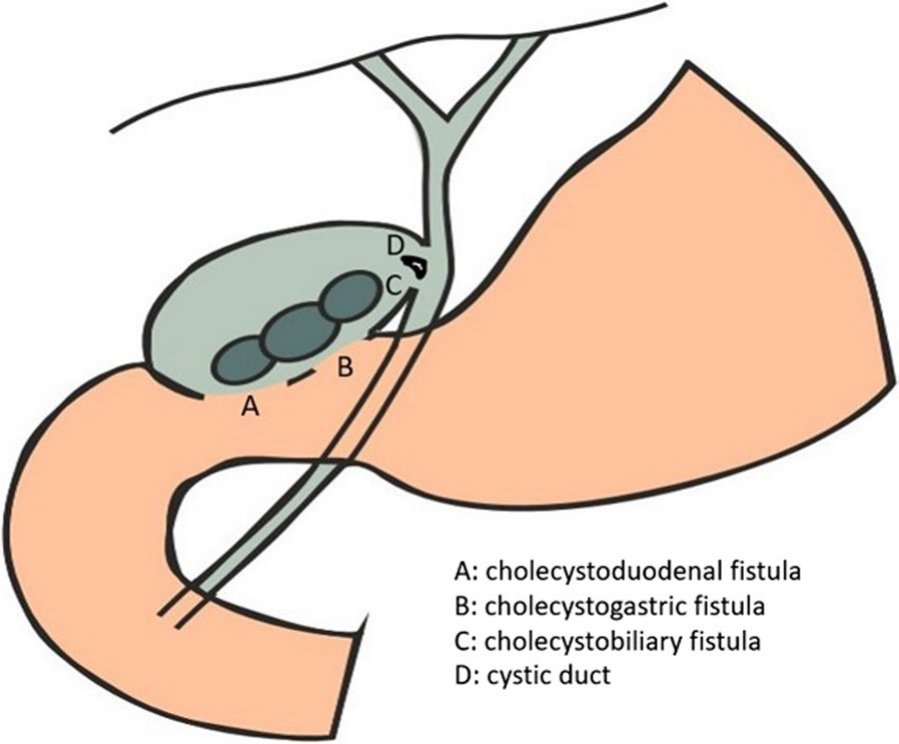
Preoperative image of gallbladder's communications with A) the duodenum, B) the pyloric antrum and C) the common bile duct. D) Site of cystic duct

**Fig. 3:**
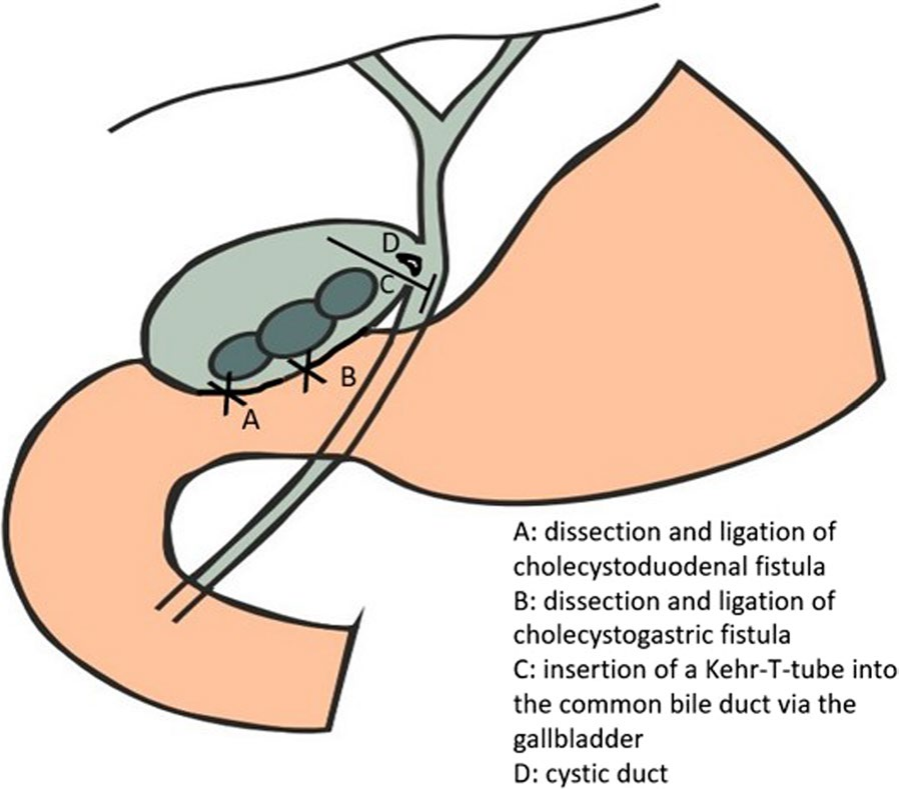
Postoperative image in which are depicted A) the site of cholecystoduodenal fistula's dissection, B) the site of cholecystogastric fistula's dissection and C) the site of Kehr-tube's insertion into the common bile duct via the gallbladder, because of the inflammation in the region. D) Site of cystic duct

Surgical treatment was immediately undergone and we decided to perform a laparotomy rather than a laparoscopic approach, because of the complications of MS and the presence of these two intricate fistulae [5, 6]. During the laparotomy procedure the findings of ERCP were confirmed, as an enlarged communication between the gallbladder and the pyloric antrum was identified, as well as, a second one, between the gallbladder and the duodenum. In addition, a third communication between the gallbladder and the common bile duct was identified with a defect of common bile duct bigger than the 2/3 of the diameter (Fig. 2). A stapler was used for the communications of gallbladder with the pyloric antrum and duodenum to ligate and dissect them. Because of the intense inflammation in the area, the effort of a Roux-en-Y anastomosis for the common bile ducts defect was judged to be unsuitable, as the risk of anastomotic disruption was really high. Due to this occasion the insertion of a Kehr T-tube into the common bile duct was decided via the gallbladder [7, 8] (Fig. 3).

After 3 months, the Kehr T-tube was removed and in the subsequent 2 years of follow-up the patient was presented without complications.

## Discussion

The MS bears its name in honor of Pablo Louis Mirizzi, a professor of Surgery, who described it first in 1948 as “Functional Hepatic Syndrome” [9]. First, the term “benign obstructive jaundice” was described, because of the external compression of the common hepatic duct from an impacted gallstone at the Hartmann’s pouch or at the cystic duct [10, 11]. Later, was noted that the inflammatory process leads to a progressive communication between the gallbladder and the CHD or the bile duct, due to necrosis of their wall [12]. Inflammatory communications of gallbladder with parts of the gastrointestinal tract such as with the stomach, the duodenum or the colon have been recognized and classified by Beltran et al. According to this classification the presence of cholecystoenteric fistula with or without gallstone ileus has been described as Type V (Va or Vb, respectively) MS [13] (Table 1). In our case, the patient diagnosed with Mirizzi Syndrome Type Va.

**Table 1:** Modified classification of Mirizzi syndrome

Type	Description
I	Extrinsic compression of the common bile duct by an impacted gallstone
II	Cholecystobiliary fistula secondary to an eroded gallstone involving 1/3 of the circumference of the common bile duct
III	Cholecystobiliary fistula involving 2/3 of the circumference of the common bile duct
IV	Cholecystobiliary fistula comprising the whole circumference of the common bile duct
V	Any type plus a cholecystoenteric fistula
Va	Without gallstone ileus
Vb	With gallstone ileus

At this moment, MRCP is the preferred method for the preoperative diagnosis of MS, as it is a non-invasive imaging technique [14, 15]. It can detect all of the special characteristics of MS, such as the presence of an impacted stone in the Hartmann’s pouch, the external compression and dilatation of the common hepatic duct, as well as the normal sized common bile duct [16, 17]. The inflammatory process of MS can be identified by the MRCP. Therefore, this diagnostic tool can differentiate this syndrome by other biliary conditions, such as cancer [18]. However, it is not suitable for localization of a cholecystoenteric fistula [19, 20].

Instead of the MRCP, ERCP is an invasive diagnostic approach, but it is considered as the gold standard method for the diagnosis of MS, as it offers a superior visualization of extra-hepatic ducts [21]. Furthermore, ERCP can accurately detect the complicated type V MS and localize a cholecystoenteric fistula [22, 23]. It can be also accompanied by therapeutic decompression by papillotomy and stent or nasal bile drainage (NBD), which allows the outcome of surgical treatment to be assessed through endoscopic NBD cholangiography [24, 25].

Surgical management is the mainstay treatment for MS, and the surgical approach varies according to each case [26]. The safest approach to manage MS Type V is always laparotomy since it has the advantage of better visualization, haptic feedback and gallbladder calculus removal before cholecystectomy and the best results were observed after a cholecystectomy and Roux-en-Y hepaticojejunostomy [27, 28]. As we mentioned above in our case the effort of a Roux-en-Y anastomosis was unsuitable and we decided to insert a Kehr T-tube into the bile duct to decompress the bile duct as well as to shape the duct and also it could be beneficial to avoid anastomotic leakage to such cases [29, 30]. After the removal of the tube no relapse of the symptoms were observed, leading to the conclusion that our treatment approach was beneficial for the patient.

## Conclusion

Mirizzi syndrome Type V with double fistula has been described in the past. However, in all these cases, one or two fistulas are presented. MS complicated with triple fistula is an extreme rare case, first described in the international literature, to the best of our knowledge, and confirms its long natural history of inflammation.

## Data Availability

Not applicable.

## Abbreviations

MS: Mirizzi syndrome
CBD: Common bile duct
CHD: Common hepatic duct
CT: Computed tomography
ERCP: Endoscopic retrograde cholangiopancreatography
MRCP: Magnetic resonance cholangiopancreatography
NBD: Nasal bile drainage
